# Persistent reshaping of cohesive sediment towards stable flocs by turbulence

**DOI:** 10.1038/s41598-023-28960-y

**Published:** 2023-01-31

**Authors:** Minglan Yu, Xiao Yu, Ashish J. Mehta, Andrew J. Manning, Faisal Khan, S. Balachandar

**Affiliations:** 1 Department of Civil and Coastal Engineering, University of Florida, Gainesville, USA; 2 Department of Mechanical and Aerospace Engineering, University of Florida, Gainesville, USA; 3 HR Wallingford, Wallingford, UK; 4 Department of Chemical Engineering, Texas A&M University, College Station, USA

**Keywords:** Physical oceanography, Fluid dynamics

## Abstract

Cohesive sediment forms flocs of various sizes and structures in the natural turbulent environment. Understanding flocculation is critical in accurately predicting sediment transport and biogeochemical cycles. In addition to aggregation and breakup, turbulence also reshapes flocs toward more stable structures. An Eulerian–Lagrangian framework has been implemented to investigate the effect of turbulence on flocculation by capturing the time-evolution of individual flocs. We have identified two floc reshaping mechanisms, namely breakage-regrowth and restructuring by hydrodynamic drag. Surface erosion is found to be the primary breakup mechanism for strong flocs, while fragile flocs tend to split into fragments of similar sizes. Aggregation of flocs of sizes comparable to or greater than the Kolmogorov scale is modulated by turbulence with lower aggregation efficiency. Our findings highlight the limiting effects of turbulence on both floc size and structure.

## Introduction

Clay mineral particles can bind with other particles, organic/inorganic compounds, bacteria, algae, etc., through both physical^1,2^ and biological^3^ cohesion to form larger and complex aggregates, known as flocs. Flocs can absorb contaminants, e.g., heavy metals and nutrients, which are of great concern to water quality^4,5^. Weathered oil can mix with fine sediment and organic particles to form sediment-oil-agglomerates that cause shoreline and seafloor contamination after major oil-spills^6–9^. The morphology of river deltas is found to exhibit distinguished variations driven by differences in the degree of sediment cohesion^10^. The accumulation of flocculated sediment in tidal marshes and mangrove forests can significantly affect the dynamics of ecosystem^11–13^.

The settling velocity is the single most important quantity that must be accurately parameterized for reliable prediction of the fate and transport of cohesive sediment. While the settling velocity of non-cohesive grains is well understood, the same cannot be said for the settling velocity of flocs. During transport, floc behavior is dependent upon its size, structure (or shape), density etc., which are cumbersome to measure due to the fragile nature of flocs and their complex 3-dimensional structures^14–16^. Therefore, modeling flocculation dynamics is essential to deduce sediment transport budgets and provide a basis for the biogeochemical cycles. If we assume that flocs are composed of nearly identical primary particles, then each floc can be reasonably characterized by its size or number density, i.e., the number of primary particles per floc volume (${n}_{f}$), and by its structure. The latter can vary from a compact structure, in which all particles occur in a tight arrangement and the floc effectively looks like a larger particle, to a structure in which the particles are arranged in a linear array. There are several ways to quantify the floc structure, e.g., in terms of circularity, concavity, aspect ratio, radius of gyration and fractal dimension.

In flocculation dynamics under turbulent flow, three fundamental processes must be mentioned: (i) *Breakup* of a floc into two or multiple smaller fragments. It can be further classified into two types^17^: First, turbulent shear can erode smaller fragments from a larger floc that are weakly attached to the main body of the floc; Second, flocs can split into two or more fragments when subject to intense tensile stress, typically resulting in nearly equal-sized daughter flocs. (ii) *Aggregation* of multiple flocs into a larger one through collision. (iii) *Restructuring* of a floc to a new, often more stable structure^18^ with the same number of primary particles. The three processes make the size and structure of flocs highly time-dependent, changing rapidly during flocculation.

Turbulence plays an important role in all three processes mentioned above in natural environments^19–22^. It can promote the growth of floc by enhancing the collision frequency^21^ but also can break unstable flocs by turbulent eddies via shear or hydrodynamic drag^23^. Moreover, turbulent eddies can restructure flocs through repeated breakup followed by rapid regrowth^24,25^ or continuous restructuring^26–28^. While breakup and aggregation have been studied in some detail^19,22,29–31^, the mechanisms of floc reshaping by turbulence remain to be explored^32,33^. Reshaping of flocs into more stable structures increases floc strength considerably and thereby reduces the chance of subsequent breakup. Both turbulent shear and hydrodynamic drag can cause floc reshaping^23^, and the relative importance of the two mechanisms depends on the strength of particle–particle interactions and turbulence intensity. With the advance in floc measurement techniques using digital imaging tools, turbulent mixing and transport processes may be deduced from the floc size and structure measurement in the field^34,35^.

The importance of floc reshaping has been recognized in past research^27,32,33,36,37^. However, experimental observations of repeated breakage-regrowth have not been achieved due to challenges associated with following a floc within a turbulent field^38–40^. Computational observations have not been forthcoming either. A numerical framework that couples the Computational Fluid Dynamic (CFD) and Discrete Element Method (DEM) has gained considerable attention in the study of both non-cohesive and cohesive sediment transport^41,42^. A process-based model which resolves particle–particle and turbulence-particle interactions will enable one to investigate the flocculation dynamics at the particle level by tracking the time-evolution of individual flocs, including their size and structure.

The present process-based simulations are designed to achieve the following three goals: (i) Demonstrate conclusive evidence of how turbulence reshapes flocs via repeated steps of breakage-regrowth and via continuous restructuring, towards more stable floc structures. (ii) Obtain reliable statistical information characterizing the breakup and aggregation (e.g., breakup rate, aggregation efficiency) required by the widely used Population Balance Equation (PBE; Methods), and (iii) examine the effects of floc properties and turbulence on breakup and aggregation parameters.

## Results

### Flocculation in homogenous isotropic turbulence

We simulated flocculation in homogeneous isotropic turbulence by coupling the direct numerical simulation (DNS) with the DEM (Methods; for more details^43^). Two contrasting cases are presented by varying the physicochemical properties of the primary particle (specifically, stickiness) while holding the mean turbulent shear rate the same (Supplementary Table S1). They are designated as case S1 with less sticky particles and case S2 with stickier particles. In each case, the simulation was initialized with 50,000 mono-dispersed soft adhesive spheres, which were uniformly distributed in the turbulent field. The non-dimensional particle size was ${D}_{p}=0.02$, and the Reynolds number based on Taylor microscale and turbulent velocity fluctuation was 33 (see details in the Methods). Due to collision driven by turbulence, flocs start to form gradually. As breakup and aggregation balance each other, the system reaches a dynamic equilibrium^43^.

**Figure 1:**
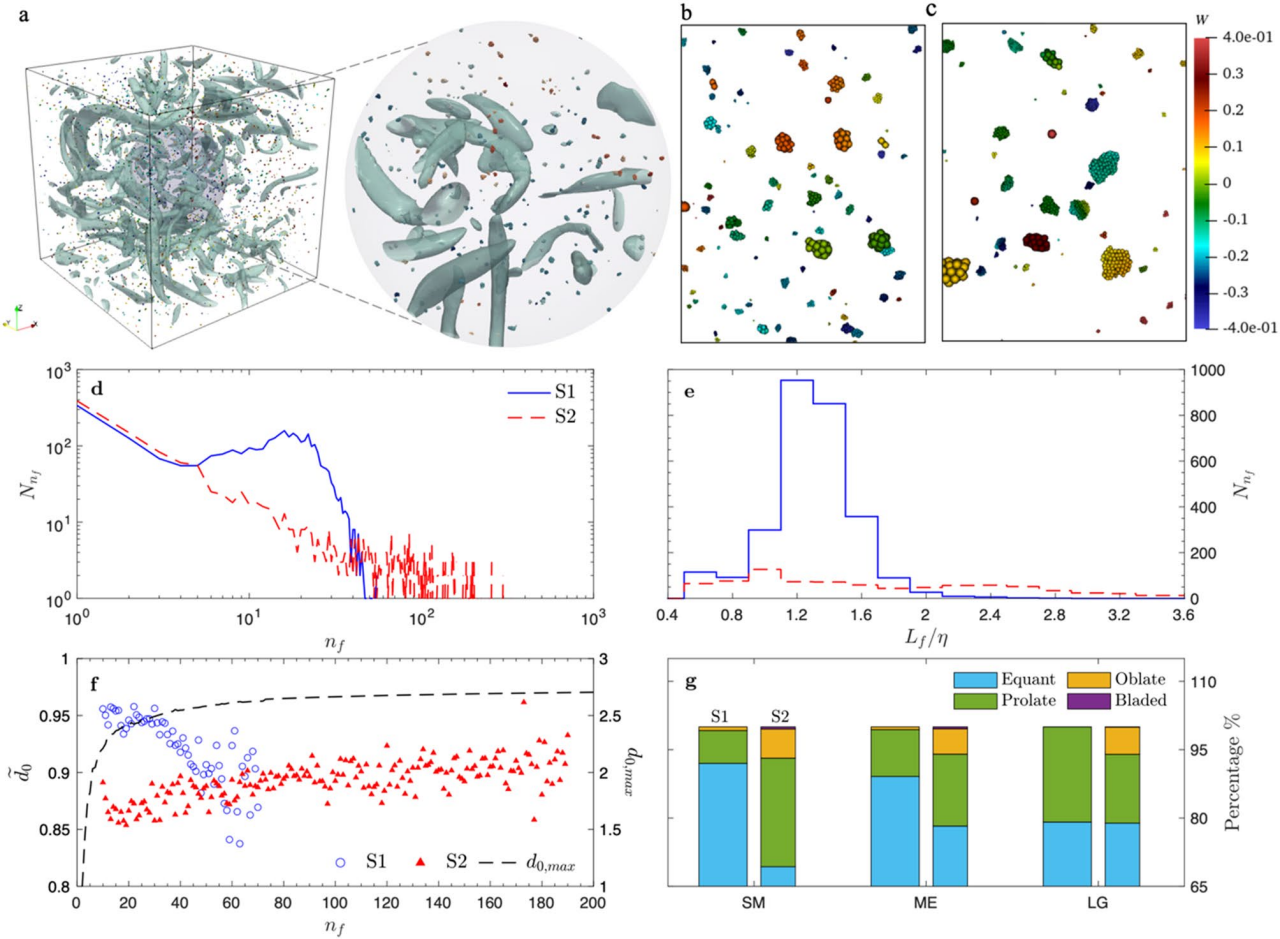
Flocs in homogenous isotropic turbulence. (**a**) Instantaneous flow field with flocs of case S1 at later stage, with a zoomed in view within a sphere of radius 2 at the domain center. The green iso-surfaces are turbulent coherent structures visualized by the $\lambda 2$ method^48^, showing the region of vortices. Particles are colored with their vertical velocity. (**b, c**) The zoom-in view shows flocs at a later stage for case S1 and S2. Small and compact flocs are generated, while larger flocs are more elongated in case S1 (**b**). In S2 (**c**), with greater particle stickiness, much larger flocs consisting of hundreds of particles are generated. Smaller flocs show irregular structures, while larger ones are much more compact. (**d, e**) Floc size distribution ${N}_{{n}_{f}}$ based on primary particle number ${n}_{f}$ and floc size ${L}_{f}$ at equilibrium. Distribution with peak around Kolmogorov length scale is observed for S1 with lower $Co$ in (**e**). Power-law size distribution is observed for case S2 with higher $Co$ in (**d**). (**f**) Normalized fractal dimension of flocs with different ${n}_{f}$ for case S1 and S2, $\widetilde{{d}_{0}}={d}_{0}/{d}_{0,max}$ (see Methods)*.* $\widetilde{{d}_{0}}$ close to 1 indicates compact floc structure. Floc structure becomes increasingly irregular and fragile as they grow in case S1. Although flocs in case S2 are more irregular in general, larger flocs show relatively compact structure and have similar fractal dimension. (**g**) Zingg’s shape classification^49^ (Supplementary Table S2) for flocs within different $n_{f}$ ranges for case S1 ($n_{f,SM} = 10 - 25, n_{f,ME} = 26 - 35$, $n_{f,LG} = 35 - 70$) and case S2 ($n _{f,SM} = 10 - 30, n_{f,ME} = 31 - 70$, $n_{f,LG} = 71 - 200$). Smaller flocs in case S1 are likely to have equant shape while those in S2 have a larger portion of prolate and oblate shapes. Large fractions of larger flocs in S1 tends to have prolate shape while large flocs in case S2 are more equant.

Floc strength relative to the turbulence intensity can be characterized by the Cohesive number, $Co=\gamma /\rho {D}_{p}k$, which is the ratio of the floc yield strength represented by the surface energy density $\gamma$ to the turbulent kinetic energy $k$. $\rho$ is the fluid density and ${D}_{p}$ is the diameter of the primary particle. The $\gamma$ of primary particles in case S1 is lower than in case S2 with respective $Co$ values of 0.54 and 5.39. We did not observe preferential local accumulation of particles in either case and flocs are nearly uniformly distributed. Figure 1a shows a representative result of case S1, where small flocs are more rounded and large flocs are more elongated (Fig. 1b). Much larger flocs with hundreds of primary particles are generated in case S2 (Fig. 1c). Interestingly, smaller flocs in case S2 present more irregular structures, while large ones are more compact.

### Macroscopic floc properties at equilibrium

During flocculation, the floc size distribution reaches equilibrium as the breakup and aggregation balance (Fig. 1d,e). For case S2 with higher $Co$, a power-law distribution can be observed for small to intermediate flocs, where the system behaves similar to a coagulation-dominated system^44^ (Fig. 1d). For case S1 with lower $Co$, a peak around ${n}_{f}$ = 18 is observed (Fig. 1d). This peak corresponds to $1.2$ times the Kolmogorov length scale ($\eta$) based on the true floc size ${L}_{f}$ (Fig. 1e; Methods), which implies the most probable floc size is limited by the smallest turbulent eddies when $Co$ is small. Similar peaks around the Kolmogorov length scale have been observed in laboratory experiments and field observations^45–47^.

As turbulence reshapes flocs continuously towards an equilibrium structure distribution, flocs consisting of different ${n}_{f}$ should have different mean equilibrium structures. We explored the mean floc structure at equilibrium using the normalized fractal dimension, $\widetilde{{d}_{0}}$ (Fig. 1f; Methods) averaged over all flocs of given ${n}_{f}$. In case S1, small flocs of ${n}_{f}<30$ are compact with $\widetilde{{d}_{0}}$ around 0.95, while larger flocs become increasingly irregular and porous with decreasing $\widetilde{{d}_{0}}$. For case S2, small flocs (${n}_{f}\approx 20$) are more irregular with lower $\widetilde{{d}_{0}}$, while they become more compact and rounded as they grow bigger ($20<{n}_{f}<100$). $\widetilde{{d}_{0}}$ reaches an asymptotic constant of 0.9 for large flocs (${n}_{f}>100$), implying their similar and compact structures. In addition, larger flocs in S1 tend to be prolate (Fig. 1g; Zingg’s shape classification^1^). In contrast, larger flocs in S2 are more spherical as the fraction of the equant shape grows with floc size, while somewhat greater fractions of smaller flocs (${n}_{f,SM}=10-30$) have prolate and oblate shapes.

### Time-evolution of floc structure

**Figure 2:**
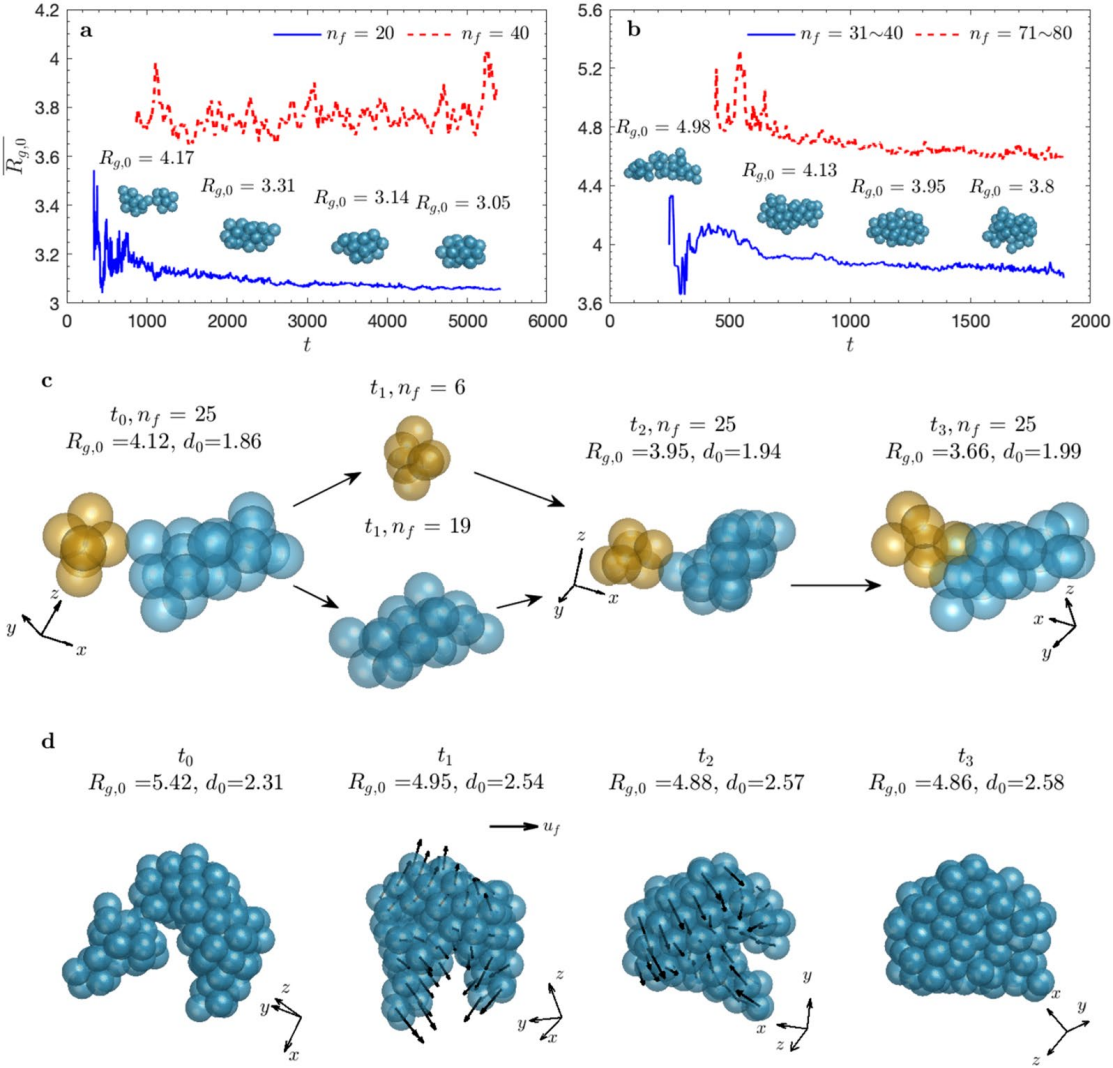
Floc reshaping by turbulence. (**a**) Time-evolution of mean radius of gyration ($\overline{{R}_{g,0}}$; see Method) of flocs consisting of 20 and 40 primary particles for case S1. Floc reshaping only takes place with smaller flocs as indicated by the decrease of $\overline{{R}_{g,0}}$ with time. Four representative flocs with ${n}_{f}=20$ at different reshaping stages are plotted to shows how the mean floc structure changes with time. (**b**) The same for S2 of flocs consisting of 31–40 and 71–80 primary particles. Floc reshaping also influences larger floc size in S2. Four representative flocs with ${n}_{f}=35$ at different stages are plotted. (**c, d**) Two floc reshaping mechanisms, namely breakage-regrowth and restructuring by hydrodynamic drag, respectively. A floc of ${n}_{f}=25$ in case S1 becomes more compact with increased contact points after several breakage-regrowth sequences in (**c**). A large floc of ${n}_{f}=102$ in case S2 goes through restructuring by hydrodynamic drag while all the particles stay connected in (**d**). The gap between two sub-flocs is closed by local converging flow indicated by the black arrows (local fluid velocity) at the particle center.

Based on the observation on floc structures at equilibrium, two interesting questions arise: Why are small flocs in case S1 more compact while those in case S2 are more irregular? How do flocs become rounded and compact? To find out, we first tracked the time-evolution of mean floc structure for flocs of given ${n}_{f}$ (Fig. 2a,b), where the mean radius of gyration ($\overline{{R}_{g,0}}$, Methods) over flocs of given ${n}_{f}$ is used. Reshaping occurs at small to intermediate floc size (${n}_{f}<40$) for S1 as indicated by decreasing $\overline{{R}_{g,0}}$ with time (Fig. 2a, blue curve). For large flocs (e.g., ${n}_{f}=40$), strong oscillations can be observed due to intermittency in turbulence (Fig. 2a, red curve). Once a porous and fragile floc with large radius of gyration (${R}_{g,0}$) forms, it is vulnerable and  can be easily broken by turbulent eddies. Floc restructuring is also evident for large flocs in S2 (Fig. 2b). They are resilient against turbulent shear due to the high particle stickiness and may be restructured by large turbulent eddies via hydrodynamic drag. The large gyration radius at early stage is due to the formation of large irregular flocs from aggregation of two or more smaller flocs of similar sizes (The first representative floc in Fig. 2a,b). These flocs are susceptible to breakup by turbulence, thus resulting in a sharp drop in $\overline{{R}_{g,0}}$.

### Evidence for different floc reshaping mechanisms

Having confirmed that flocs do adjust their structures due to turbulence during flocculation, it was natural to investigate the detailed mechanisms on how turbulence reshapes flocs of different size and cooperates with their cohesive behavior. The most fascinating phenomenon that we observe is the persistent action of turbulence in forming stable flocs through tracking the evolution of individual flocs. This process resembles a toddler shape-sorting different objects into their respective slots by persistently trying different options until the correct solution is achieved. In a somewhat similar manner, under strong enough turbulent shear, large non-compact flocs break into smaller flocs, with the latter quickly reaggregating to reform a typically compact floc^50–52^. Turbulence seems to “try” several repeated breakage-regrowth steps until a stable configuration, which cannot be broken at that level of turbulence, is reached (Fig. 2c). When viewed in this way, the continuous reshaping process can also be thought of as the limiting case of breakup-regrowth happening at a nearby location in immediate succession. When turbulent shear is moderate or weak compared to the adhesive forces holding the primary particles together in a floc, continuous restructuring occurs while the primary particles stay connected by hydrodynamic drag (Fig. 2d).

For case S1 ($Co=0.54$), breakage-regrowth dominates the reshaping of smaller flocs. Turbulent shear induced by small turbulent eddies effectively breaks flocs with irregular structures and weak bonds (i.e., low flocs strength). In Fig. 2c, at time ${t}_{0}$, two sub-flocs are bonded at a single contact point, which is the weakest link of the floc. The floc has a low fractal dimension (${d}_{0}$) of 1.86. As the floc breaks at its weakest link, two daughter flocs are produced at ${t}_{1}$. They stay close and are brought back together by the turbulent eddy at a different orientation, which results in two contact points, resulting in a higher ${d}_{0}$ of $1.94$. This process is repeated several times and the floc structure becomes more compact. For case S2 ($Co=5.39$), restructuring is the dominant mechanism in the formation of large and compact flocs. Turbulent shear due to small turbulent eddies is not strong enough to break small flocs due to large particle stickiness, allowing them to maintain more irregular floc structures compared to those in case S1. When flocs grow, large turbulent eddies start to restructure the floc through hydrodynamic drag. At ${t}_{0}$, a large gap exists in a large floc of ${n}_{f}=102$ in case S2 (Fig. 2d). The local converging flow (black arrows) brings the two arms closer between ${t}_{1}$ and ${t}_{3}$. The gap is closed later, and the floc becomes more compact and rounded with ${d}_{0}=2.58$.

### Flocculation dynamics: breakup and aggregation

The floc strength changes considerably due to floc reshaping, which in turn affects aggregation and breakup^24,36^. DEM tracks the motions of individual particles and allows one to track the aggregation and breakup of individual flocs, which is extremely difficult to capture in laboratory experiments. We now present the valuable information gathered from the present simulations that can help improve the widely used population balance model (PBM; Methods).

The kinetics of breakup in PBM is characterized by (i) the breakup rate and (ii) the size distribution of the resulting daughter flocs (breakage distribution function). Large and porous flocs have higher breakup rates. The breakage distribution function is typically defined as binary-^32,53^ or normal-distribution^50^, giving preference to equal-sized breakup^54^. The aggregation model is characterized by (i) the collision frequency and (ii) the collision efficiency. When collisions occur mainly due to fluid shear, collision frequency scales with the turbulence shear rate. Collision efficiency represents the probability of two flocs staying together after collision. It depends on the properties of particles, the hydrodynamic stress, and the floc properties such as size and structure.

### Breakup: effect of floc structure

**Figure 3:**
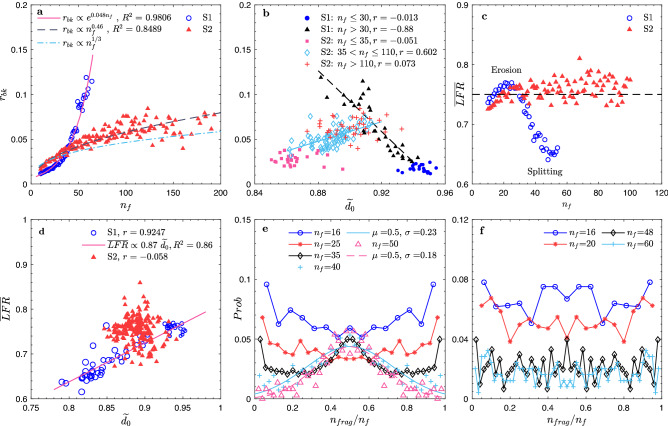
Effects of floc properties on the breakup process. Both floc size and floc structure can affect breakup, including the breakup rate and breakage distribution function. (**a**) Breakup rate ${r}_{bk}$ for flocs consisting of different number of primary particles in case S1 and S2. Power-law relation between ${n}_{f}$ and ${r}_{bk}$ is found for case S2, suggesting the independence of breakup rate on floc structure based on the empirical function ${r}_{bk}\propto {V}^{1/3}$ (which only considers the effect of floc size). ${r}_{bk}$ grows exponentially with ${n}_{f}$, as larger flocs in S1 becomes increasingly fragile. (**b**) Breakup rate ${r}_{bk}$ changes with floc structure characterized by the normalized fractal dimension $\widetilde{{d}_{0}}$. $r$ is the correlation coefficient. ${r}_{bk}$ is negatively correlated with the floc fractal dimension in case S1, while opposite relation is found in case S2, again showing the effect of structure on ${r}_{bk}$ for case S1 with lower $Co$. (**c**) The mean floc breakup mode for flocs of different sizes quantified by the largest fragment ratio $LFR$ (Methods) averaged over flocs of given ${n}_{f}$, denoted as $\overline{LFR}$. Both cases show the dominance of erosion ($\overline{LFR}$>0.75) at specific size range. (**d**) Relations between the mean floc breakup mode ($\overline{LFR}$) and the floc structure ($\widetilde{{d}_{0}}$), where larger marker size indicates larger floc size. Breakup mode is correlated with the floc structure in case S1, while it is independent of floc structure in case S2. (**e, f**) Breakage distribution function for S1 and S2. Fragment sizes are normalized by the corresponding size of the breaking floc, ${n}_{frag}/{n}_{f}$. Only data of flocs that break into two fragments are included. For ${n}_{f}=40$ and $50$ in case S1, the breakage distributions are fitted by a normal distribution in (**e**).

As there is no theory to predict the breakup rate from first principles, it is usually obtained from fitted parameters based on the floc size distribution data^54^. From our simulation results, we can calculate the breakup rate quantitatively (see Methods). For case S2 with large $Co$, the breakup rate ${r}_{bk}$ increases with ${n}_{f}$ following a power-law relation (Fig. 3a). As the structure of large flocs in S2 has a weak dependence on the floc size, the empirical formula^50,55^ ${r}_{bk}\propto {V}^{1/3}$ holds without the effect of floc structure. For S1, the breakup rate grows exponentially with ${n}_{f}$ as the floc structure becomes more irregular and fragile (Fig. 3a,b). Interestingly, while the breakup rate decreases with $\widetilde{{d}_{0}}$ in case S1, it increases with $\widetilde{{d}_{0}}$ in case S2 (Fig. 3b). For compact small flocs in case S1, ${r}_{bk}$ stays constant around 0.017 (Fig. 3b, circles). For large flocs in S1, ${r}_{bk}$ decreases linearly with $\widetilde{{d}_{0}}$, suggesting a higher breakup rate for irregular flocs (Fig. 3b, triangles). In S2, small flocs have the lowest breakup rate around 0.03 (Fig. 3b, squares). They can maintain their irregular structures with low $\widetilde{{d}_{0}}$ and are resilient against small turbulent eddies. For larger flocs, ${r}_{bk}$ increases with $\widetilde{{d}_{0}}$ as large turbulent eddies start to interact with them (Fig. 3b, diamonds), indicating the dominant effect of floc size. For even larger flocs, ${r}_{bk}$ remains constant around 0.07, independent of floc structure (Fig. 3b, pluses).

Each breakup event can be classified into either erosion or splitting, using the largest fragment ratio ($LFR$; see Methods). *LFR* is averaged over flocs of the same ${n}_{f}$ to investigate the mean breakup mode for flocs of different sizes, denoted as $\overline{LFR}$ (Fig. 3c). A threshold value of 0.75 is used to determine the relative importance of the two modes. In S1, $\overline{LFR}$ is slightly greater than 0.75 for flocs with ${n}_{f}$ between 16 and 25, suggesting both modes are present, but erosion is preferred. For flocs with ${n}_{f}>30$, $\overline{LFR}$ drops from 0.75 to around 0.65, suggesting splitting becomes more frequent as flocs become more fragile. For S2, $\overline{LFR}$ increases slightly from 0.74 to 0.76 in the range of $16\le {n}_{f}\le 25$, then remains constant around 0.77 as flocs grow further, showing a slight prevalence of erosion for compact flocs. A linear relation can be observed in case S1 with less sticky primary particles, where $\overline{LFR}$ increases with $\widetilde{{d}_{0}}$ (Fig. 3d). Compact flocs tend to erode, while irregular flocs with low $\widetilde{{d}_{0}}$ tend to split when they break. However, the correlation between $\overline{LFR}$ and $\widetilde{{d}_{0}}$ is weak for S2*,* suggesting the breakup mode and floc structure are independent for strong compact flocs.

The breakage distribution function is further investigated, where the fragment size is normalized by the size of the parent floc as ${n}_{frag}/{n}_{f}$ (Fig. 3e,f). Peaks at two ends indicate erosion, while a peak at the center suggests splitting. In case S1 with smaller $Co$ (Fig. 3e), the breakage distribution function changes from an erosion dominant shape when ${n}_{f}\le 25$ to a splitting dominant (normal distribution) shape when ${n}_{f}\ge 40$. For the intermediate floc size of ${n}_{f}=35$, both two modes are equally important, with two peaks at both ends and one in the middle. For case S2, both modes are important when smaller flocs (e.g., ${n}_{f}=16$) break up in turbulent flow (Fig. 3f). The distribution function then changes to an erosion dominant shape as the floc size grows (e.g., ${n}_{f}=20$). For flocs of ${n}_{f}=48, 60$, the daughter floc size is more uniformly distributed. Again, we observe the relative prevalence of the erosion mode.

### Aggregation: effects of pre-collision floc properties and turbulent modulation

**Figure 4:**
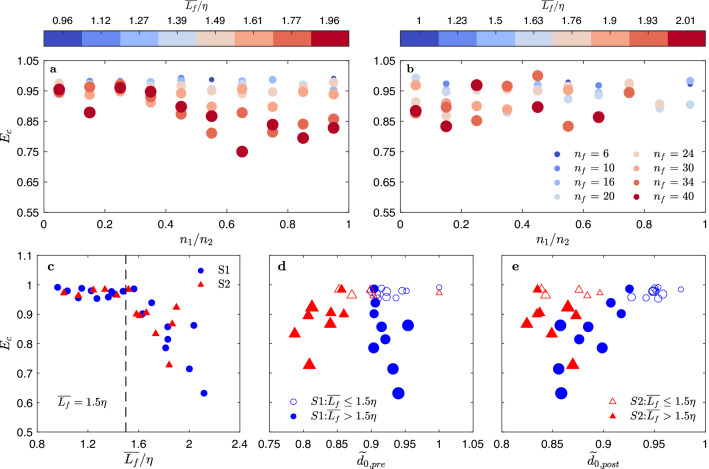
The effects of floc properties and turbulent modulation on the aggregation efficiency. (**a, b**) Dependence of aggregation efficiency (${E}_{c}$) on the pre-collision floc size ratio (${n}_{1}/{n}_{2}<1$) for all aggregation resulting in given ${n}_{f}$. The resultant floc size over the Kolmogorov length scale ($\overline{{L}_{f}} /\eta$) is indicated by the color bar. For flocs of ${n}_{f}\le 24$ or $\overline{{L}_{f}}<1.5\eta$ in case S1 (see **a**) and all flocs in case S2 (see **b**), ${E}_{c}$ is independent of to the floc size ratio before aggregation. For large flocs of $\overline{{L}_{f}}>1.5\eta$ in case S1 (see **a**), ${E}_{c}$ is high when two flocs of very different size (${n}_{1}/{n}_{2}\sim 0$) collide, while ${E}_{c}$ is low for the collision of two equal-sized flocs (${n}_{1}/{n}_{2}\sim 1$). (**c**) Relation between the aggregation efficiency ${E}_{c}$ and the resultant normalized floc size ($\overline{{L}_{f}}/\eta$) of equal-sized collision with ${n}_{1}/{n}_{2}\sim 1$. ${E}_{c}$ decreases when the post-collision floc size $\overline{{L}_{f}}>1.5\eta$. (**d, e**) Relation between aggregation efficiency ${E}_{c}$ and the pre- and post-collision floc structures (characterized by the normalized fractal dimension $\widetilde{{d}_{0}}$), when two flocs with equal ${n}_{f}$ collide. For aggregation generates flocs smaller than $1.5\eta$ (hollow markers), ${E}_{c}$ is high for both cases and is independent of both the pre- and post-collision floc structures. For aggregation that results in flocs larger than $1.5\eta$ (see **d**, filled markers), ${E}_{c}$ decreases as pre-collision flocs become larger and compact in case S1, while as pre-collision flocs becomes larger and irregular in case S2. When two equal-sized flocs of increasingly compact structure aggregate in S1 (see **d**, filled blue circle), the resultant larger flocs become increasingly irregular with decreasing fractal dimension(see **e**, filled blue circle). When two equal-sized flocs of more irregular structure aggregate in case S2 (see **d**, filled red triangle), the resultant larger floc is more compact with increasing fractal dimension (see **e**, filled red triangle).

Due to the complexity of estimating collision efficiency, it is often treated as a constant, or as a fitted parameter in models^55,56^. In flocculation models based on PBE, the formula of collision efficiency is typically scaled with the pre-collision floc size ratio (${L}_{i}/{L}_{j}, {L}_{i}<{L}_{j}$)^57,58^. Similarly, we looked at the effect of size ratio based on the primary particle number, defined as ${n}_{1}/{n}_{2}$ (${n}_{1}<{n}_{2}$), on the aggregation efficiency to generate larger flocs of given ${n}_{f}$ (Fig. 4a,b). In S1, the aggregation efficiency (${E}_{c}$; see Methods) is always high for aggregation forming flocs of ${n}_{f}\le 24$ or $\overline{{L}_{f}}<1.5\eta$, regardless of the pre-collision floc size ratio (Fig. 4a). When the resultant flocs are large (e.g., ${n}_{f}=30, 34, 40$ or $\overline{{L}_{f}}>1.5\eta$), ${E}_{c}$ drops with increasing pre-collision floc size ratio, suggesting lower efficiency when two equal-sized flocs collide (${n}_{1}\approx {n}_{2}$). However, the effect of size ratio is not evident in case S2 with higher $Co$ (Fig. 4b). The turning point around $1.5\eta$ implies the modulation by turbulence. Smaller flocs may not “feel” the shear effect by turbulent eddies, and thus have high ${E}_{c}$. As the floc size is comparable to or larger than the Kolmogorov length scale, floc can be broken more easily by turbulent eddies, resulting in lower ${E}_{c}$.

The pre-collision floc properties (size and structure) and the way two or more flocs attach to each other tend to determine the post-collision floc properties and affect the aggregation efficiency. We studied the case when two equal-sized flocs (${n}_{1}={n}_{2}$) aggregate, particularly to explore the effect of pre- and post-collision floc structures (pre-structure and post-structure) on the aggregation efficiency (Fig. 4c–e). ${E}_{c}$ stays high when the post-aggregation floc size (post-size) is smaller than $1.5\eta$, and is independent of both the pre- and post-structure (Fig. 4d,e). The aggregation efficiency for post-size greater than $1.5\eta$ drops sharply in both cases as the turbulent eddies start to play a major role in breaking them, again showing the modulation by turbulent eddies by limiting the size of newly formed flocs^59^.

Surprisingly, the pre- and post-structure play different roles in the two cases in collisions that lead to a floc of size greater than $1.5\eta$. For case S1, ${E}_{c}$ decreases with increasing pre-collision $\widetilde{{d}_{0}}$ for aggregation with post-size ${L}_{f}>1.5\eta$ (Fig. 4d, filled circle). Larger flocs made of two compact equal-sized flocs exhibit irregular and fragile structures with lower fractal dimension (Fig. 4e, filled circle), which can be easily broken by turbulent eddies. On the contrary, for case S2, ${E}_{c}$ continues to decrease as the two aggregating flocs become larger and more irregular with decreasing $\widetilde{{d}_{0}}$ (Fig. 4d, filled triangle). In the meantime, the post-structure becomes more compact with increasing $\widetilde{{d}_{0}}$ (Fig. 4e, filled triangle). The compact post-structure makes flocs more resilient to turbulent shear. However, the efficiency keeps decreasing as the post-structure becomes more compact, suggesting the floc size controls the aggregation efficiency in S2. The observation of two irregular flocs forming a compact floc indicates the two aggregating flocs are likely to attach to each other with their minor axis (shortest dimension) aligned.

## Conclusion

Flocs generated under turbulence would go through persistent reshaping toward more compact and stable structures. Compaction of flocs has been observed as a reduction in the mean radius of gyration with time, which eventually approaches an asymptotic value (Fig. 2a,b). Two floc reshaping mechanisms, namely breakage-regrowth and restructuring by hydrodynamic drag, have been identified and documented. Their relative importance strongly depends on the Cohesion number (*Co*) defined as the ratio of floc strength to the turbulent intensity. At low *Co*, turbulent shear is effective in breaking fragile flocs, and the breakage-regrowth mechanism dominates floc reshaping (Fig. 2c). At large *Co*, turbulent shear by small-scale turbulent eddies is not strong enough to break the flocs. On the other hand, hydrodynamic drag due to turbulent eddies of comparable size to the floc can restructure the flocs, in which primary particles change their relative position but stay in contact (Fig. 2d).

Breakup is also affected by the floc reshaping and strongly depends on *Co*. Both floc size and structure were found to influence the breakup rate at low *Co*. In addition, the breakup mode is highly dependent on the floc structure. Compact and rounded flocs are more likely to be eroded by turbulent eddies, whereas irregular flocs are more likely to split into daughter flocs of comparable sizes. The corresponding breakage distribution function changes from one with erosion dominant shape to a normal distribution (splitting dominant) as flocs become larger and more fragile. However, at large *Co*, as flocs are resilient to turbulent shear, floc size is the key factor when estimating the breakup rate. Erosion is more prevalent regardless of floc structure. In general, the formula of breakup rate should be adjusted based on the *Co*. A breakage distribution function gives preference to erosion should be considered, and different breakage distribution functions may be required for flocs of different sizes in the implementation of PBE simulation for more accurate predictions.

In turbulent environment, the aggregation efficiency (${E}_{c}$) is modulated by turbulence via a reduction in the probability of generating floc of ${L}_{f}>1.5\eta$. Those larger flocs can be easily broken by turbulent eddies. The pre-collision floc size ratio is only found to affect ${E}_{c}$ for case of low *Co* when the post-collision size ${L}_{f}>1.5\eta$. ${E}_{c}$ is higher when two flocs of different sizes collide and is lower when two flocs of similar sizes collide. The collision of two compact equal-sized flocs can produce larger flocs of more irregular and fragile structures. When two irregular equal-sized flocs collide, they are likely to align along their minor axes and produce a more compact floc. As only two cases are presented here, it is not feasible to deduce a general formula describing aggregation efficiency. Our results highlight the significance of the limiting effect of turbulent eddies not only on floc size but also on floc shape.

## Methods

### Discrete element method with soft adhesive spheres

We simulated particle–particle interactions using the Discrete Element Method (DEM), which solves for the translational motion for all particles based on force balance:

1 $$\begin{array}{*{20}c} {m\frac{{d{\varvec{v}}}}{dt} = {\varvec{F}}_{hz} + {\varvec{F}}_{vdw} + {\varvec{F}}_{JKR} + {\varvec{F}}_{hydro} ,} \\ \end{array}$$

in which ${\varvec{v}}$ is the particle velocity, $m$ is the mass of the particle, ${{\varvec{F}}}_{hz}$, ${{\varvec{F}}}_{vdw}$, ${{\varvec{F}}}_{JKR}$ and ${{\varvec{F}}}_{hydro}$ are the Hertzian contact force, van der Waals force, adhesive contact force based on Johnson-Kendall-Roberts (JKR) theory, and hydrodynamic force acting on the particle, respectively. Cohesive particles are modeled as soft adhesive spheres, which allows small overlaps between particles. Collisions among particles are modeled by the Hertzian contact model:

2 $$\begin{array}{*{20}c} {{\varvec{F}}_{hz} = \sqrt \delta \sqrt {\frac{{R_{i} R_{j} }}{{R_{i} + R_{j} }}} \left[ {\left( {k_{n} \delta {\varvec{n}}_{ij} - m_{eff} \gamma_{n} {\varvec{v}}_{n} } \right) - m_{eff} \gamma_{t} {\varvec{v}}_{t} } \right],} \\ \end{array}$$

where $\delta =\left({R}_{i}+{R}_{j}\right)-\left|{{\varvec{x}}}_{i}-{{\varvec{x}}}_{j}\right|$ is the overlap distance between two particles of radius ${R}_{i}$ and ${R}_{j}$, located at ${{\varvec{x}}}_{i}$ and ${{\varvec{x}}}_{j}$, respectively. ${k}_{n}$ is the elastic constant for normal contact. ${\gamma }_{n}$ and ${\gamma }_{t}$ are the viscoelastic damping constant for normal contact and tangential contact. Note the tangential contact only includes the damping term. ${m}_{eff}={m}_{i}{m}_{j}/({m}_{i}+{m}_{j})$ is the effective mass of two particles of mass ${m}_{i}$ and ${m}_{j}$. ${{\varvec{v}}}_{n}$ is the normal component of the relative velocity of two particles, and ${{\varvec{v}}}_{t}$ is the tangential component. The normal push-back force of two overlapping spherical particles is proportional to the overlap area, hence it is a nonlinear function of the overlap distance $\delta$. When two adhesive particles are in contact with each other, the adhesive force binds them together. It is modeled by the JKR theory as:

3 $$\begin{array}{*{20}c} {{\varvec{F}}_{JKR} = 4\sqrt {\frac{{\pi a^{3} \gamma_{JKR} E}}{{2\left( {1 - \nu_{p}^{2} } \right)}}} {\varvec{n}}_{ij} ,} \\ \end{array}$$

where ${\gamma }_{JKR}$ is the surface energy density, *a* is the contact radius to the deformation, *E* is the Young’s Modulus, and ${\nu }_{p}$ is the Poisson ratio of the particle.

The cohesive force is also included to model cohesive sediment particles. We used the Derjaguin–Landau–Verwey–Overbeek (DLVO) theory to model the short-range cohesive force when particles interacting with each other without physical contact. The interaction force between two particles is the sum of the attractive (van der Waals) force and the repulsive (electrostatic) force. The electrostatic force does not significantly influence particle cohesion and is often neglected in cohesive sediment studies. The van der Waals force is modeled as:

4 $$\begin{array}{*{20}c} {{\varvec{F}}_{vdw} = \left\{ {\begin{array}{*{20}l} {\frac{{A_{H} R_{eff} }}{{6D_{{{\text{min}}}}^{2} }}{\varvec{e}}_{n} ,} \hfill & {\text{if}\quad \delta > 0} \hfill \\ {\frac{{A_{H} R_{eff} }}{{6\left( {\delta - D_{{{\text{min}}}} } \right)^{2} }}{\varvec{e}}_{n} ,} \hfill & {\text{if}\quad - D_{{{\text{max}}}} \le \delta < 0} \hfill \\ {0,} \hfill & {\text{if}\quad \delta < - D_{{{\text{max}}}} } \hfill \\ \end{array} } \right. ,} \\ \end{array}$$

where ${A}_{H}$ is the Hamaker constant, which is related to the surface energy density ${\gamma }_{{A}_{H}}$ as ${A}_{H}=24\pi {D}_{\mathrm{min}}^{2}{\gamma }_{{A}_{H}}$. ${D}_{\mathrm{min}}$ is introduced to avoid singularity when $\delta$ = 0 and can be interpreted as the surface roughness of the particle. ${D}_{\mathrm{max}}$ is the cutoff distance of the van der Waals interaction, beyond which the interaction is negligible.

The time step for DEM simulations is inversely proportional to the Young’s Modulus of the particle, which often results in extremely small time-step. In practice, the Young’s Modulus is reduced by several order of magnitude without affecting the bulk dynamics of the system. For cohesive sediment studies, both the adhesive and cohesive forces need also to be scaled to guarantee the relative importance of collisional stress, adhesive and cohesive forces do not change.

In this study, only the hydrodynamic drag force (${{\varvec{F}}}_{d}$) and the buoyancy force (${F}_{b}$) are included and the hydrodynamic force on the particle is ${{\varvec{F}}}_{hydro}={{\varvec{F}}}_{d}+{{\varvec{F}}}_{b}.$ The inertial force is negligible due to small particle response time (or Stokes number). Following Wen’s drag model^60^, the drag force on the particle is given as

5 $$\begin{array}{*{20}c} {{\varvec{F}}_{d} = \frac{1}{2}A_{p} \rho C_{D0} \phi_{f}^{ - 2.7} \left| {{\varvec{u}} - {\varvec{v}}} \right|\left( {{\varvec{u}} - {\varvec{v}}} \right),} \\ \end{array}$$

where ${A}_{p}=\pi {D}^{2} /4$ is the projected area of the particle of diameter ${D}_{p}$, ${\phi }_{f}$ is the volume fraction of the fluid phase, and the drag coefficient ${C}_{D0}$ is given by

6 $$\begin{array}{*{20}c} {C_{D0} = \left\{ {\begin{array}{*{20}l} {\frac{24}{{{\text{Re}}_{p} }}\left( {1 + 0.15{\text{Re}}_{p}^{0.687} } \right),} \hfill & {\text{if}\quad {\text{Re}}_{p} < 1000} \hfill \\ {0.44,} \hfill & \text{otherwise} \hfill \\ \end{array} } \right.,} \\ \end{array}$$

where ${\mathrm{Re}}_{p}=\frac{\left|{\varvec{u}}-{\varvec{v}}\right|{D}_{p}}{\nu }$ is the particle Reynolds number and $\nu$ is the kinematic viscosity of the fluid. The buoyancy force is directly applied on the particle as

7 $$\begin{array}{*{20}c} {{\varvec{F}}_{b} = - \rho \varvec{g}V_{p} ,} \\ \end{array}$$

where ${\varvec{g}}$ is the gravitational acceleration and ${V}_{p}$ is the particle volume.

### One-way coupling of the continuous fluid and the dispersed sediment phase

Euler–Lagrangian two-phase model framework was implemented to model interactions between turbulent eddies and sediment. The fluid cell is larger than the size of the primary particle, flows around individual particles are not resolved. Since the volumetric sediment concentration is around 0.04%, the flow can be treated as dilute. The one-way coupling only considers the effect of fluid on the particle was implemented^61^. The governing continuity and momentum equations are as follow:

8 $$\begin{array}{*{20}c} {\nabla \cdot \varvec{u} = 0,} \\ \end{array}$$

9 $$\begin{array}{*{20}c} {\frac{{\partial {\varvec{u}}}}{\partial t} + \varvec{u} \cdot \nabla \varvec{u} = - \frac{1}{\rho }\nabla p + \nabla \cdot \tau + \alpha \varvec{u}. } \\ \end{array}$$

The last term is the linear forcing term that was used to drive the isotropic turbulence^62^. The above equations are solved in non-dimensional forms.

### Numerical setup and simulation parameters

We solve the governing equations in a non-dimensional form. The fluid phase was modeled using Direct numerical simulations (DNS) with the code Nek5000^63^. The computational domain was $8\times 8\times 8$ and periodic boundary conditions were applied in each direction. The average grid size is 0.067, which is greater than the particle diameter$.$ A fixed time step was chosen, ensuring a maximum Courant–Friedrichs–Lewy (CFL) number of 0.3. The mean turbulent shear rate is around $G$ = 1.6 and the Kolmogorov length scale is $\eta$ = 0.053. For homogeneous isotropic turbulence, the Reynolds number ($R{e}_{\lambda }=\lambda {u}_{rms}/\nu$= 33) based on the average Taylor microscale ($\lambda$) and turbulent velocity fluctuation (${u}_{rms}$) was used to characterize the turbulence. The non-dimensional particle size is ${D}_{p}$ = 0.02. Initially, 50,000 spherical particles were uniformly placed in the domain, leading to a volumetric sediment concentration of $\phi =4.09\times {10}^{-4}$. In this study, the sediment is quartz-based with a specific gravity of 2.65.

Model results can be interpreted in the dimensional form with given a characteristic length scale and a velocity scale. Due to the limitation of computational resources, the present study focuses on energetic environments at relatively large ${\mathrm{Re}}_{\lambda }$. By choosing a characteristic length scale of $L={10}^{-3}m$ and a characteristic velocity scale of $U$ = 0.2 m/s, the primary particle diameter was 20 $\mathrm{\mu m}$, the Kolmogorov length scale was 53 $\mathrm{\mu m}$ and the shear rate was 320 ${\mathrm{s}}^{-1}$. The particles can be interpolated as the smallest clay-based aggregates, floculli, which seldom break down to the lowest-level primary particles even at high turbulent shear^64,65^. The large turbulent shear could occur in high-energy estuaries and near-field river plumes.

### Particle number per floc or floc mass

In our simulation, each floc consists of ${n}_{f}$ number of identical spherical primary particles. Only small overlaps are allowed between particles during contact. For flocs with the same ${n}_{f}$, the corresponding floc mass (${m}_{f}$) and volume ${(V}_{f})$ would be similar regardless of the floc structure:

10 $$\begin{array}{*{20}c} {m_{f} = \rho_{s} V_{f} = \frac{\pi }{6}\rho_{s} n_{f} D_{p}^{3} }, \\ \end{array}$$

where ${\rho }_{s}$ is the particle density, ${D}_{p}$ is the diameter of primary particle. Therefore, the use of ${n}_{f}$ is equivalent to the floc mass or volume.

### Definition of floc size

For a three-dimensional floc, there are several ways to define its representative length scale, such as the volumetric (or nominal) diameter, perimeter diameter, surface diameter. To better describe the floc size, we treated each floc as a point set represented by the particle center. The Principal Component Analysis (PCA) is applied to obtain three principal axes, and the floc size along each axis is labeled as ${L}_{a}$, ${L}_{b}$ and ${L}_{c}$ in descending order. We use the longest dimension as the characteristic floc size ${{L}_{f}=L}_{a}$.

### Normalized fractal dimension

Flocs are commonly modeled as fractal entities characterized by fractal dimension ${d}_{0}$, which is defined as^66^

11 $$\begin{array}{*{20}c} {n_{f} = \left( {\frac{{L_{f} }}{{D_{p} }}} \right)^{{d_{0} }} ,} \\ \end{array}$$

in which ${D}_{p}$ is the diameter of the primary particle. Rod- or string-like flocs have fractal dimension of 1 and spherical flocs have fractal dimension of 3. The above equation works well for flocs with large number of primary particles. For flocs consisting of limited number of primary particles, the fractal dimension also depends on the ${n}_{f}$ due to finite possible configurations. For instance, two particles can only form a rod-like structure with ${d}_{0}=1$. For trimers with three identical particles, the most compact structure is an equilateral triangle giving ${d}_{0}=1.58$, while the most aspherical structure is still a rod with ${d}_{0}=1$. To account for the effect of finite number of primary particles, ${d}_{0}$ is normalized by its maximum value ${d}_{0,\mathrm{max}}$, which is based on the most compact structure of floc of ${n}_{f}$ primary particle:

12 $$\begin{array}{*{20}c} {\widetilde{{d_{0} }} = d_{0} /d_{{0,{\text{max}}}} .} \\ \end{array}$$

In general, ${d}_{0,max}$ can be computed based on the solid floc volume fraction in bounding space, ${\phi }_{floc}$ as

13 $$\begin{array}{*{20}c} {d_{{0,{\text{max}}}} = 3\left( {1 + \frac{{\ln (\phi_{floc} )}}{{\ln \left( {n_{f} } \right) - \ln \left( {\phi_{floc} } \right)}}} \right).} \\ \end{array}$$

For small to intermediate flocs (${n}_{f}\le 72$), ${\phi }_{floc}$ values of sphere packing in sphere are used^67^. For large flocs (${n}_{f}>72$), random loose packing of ${\phi }_{floc}=0.56$ is used. A $\widetilde{{d}_{0}}$ value close to 1 shows the floc has a structure close to its most compact arrangement, while $\widetilde{{d}_{0}}$ close to 0 suggests irregular and aspherical structure.

### Radius of gyration of a floc of identical spheres

The radius of gyration is defined as the root-mean-square distance of particles from the floc center as

14 $$\begin{array}{*{20}c} {R_{g} = \sqrt {\frac{1}{{n_{f} }}\sum\nolimits_{i = 1}^{{n_{f} }} {\left| {{\varvec{x}}_{i} - {\varvec{x}}_{c} } \right|^{2} } } ,} \\ \end{array}$$

where ${{\varvec{x}}}_{i}$ is the position of the ${i}^{th}$ primary particle in the floc and ${{\varvec{x}}}_{c}$ is the floc center. The normalized radius of gyration is ${R}_{g,0}=({R}_{g} +1)/{R}_{p}$, where ${R}_{p}$ is the radius of particle.

### Calculation of breakup rate

The floc breakup rate ${r}_{bk}$ for a floc of given ${n}_{f}$ is defined as the ratio of number of flocs that undergo breakup in a fixed time interval (${N}_{{n}_{f},bk}$) to the total number of flocs (${N}_{{n}_{f}}$) of that given size as

15 $$\begin{array}{*{20}c} {r_{bk} \left( {n_{f} } \right) = N_{{n_{f} ,bk}} /N_{{n_{f} }} .} \\ \end{array}$$

To find breaking flocs, we tracked each floc over one output time step ($t\sim t+\Delta t$). Based on the change of primary particles within flocs, three floc states can be identified: (i) flocs stay unchanged, ${n}_{f}\left(t\right)={n}_{f}\left(t+\Delta t\right)$; (ii) flocs gain additional particles by aggregation, ${n}_{f}\left(t\right)<{n}_{f}(t+\Delta t)$; (iii) flocs lose particles by breakup, ${n}_{f}\left(t\right)>{n}_{f}\left(t+\Delta t\right)$. The number of flocs from the three groups of given ${n}_{f}$ are named as ${N}_{{n}_{f},uc}$, ${N}_{{n}_{f},ag}$ and ${N}_{{n}_{f},bk}$, respectively. The total number of flocs at each output time (${N}_{{n}_{f}}$) is the sum of the number of flocs from each group,

16 $$\begin{array}{*{20}c} {N_{nf} = N_{{n_{f} ,uc}} + N_{{n_{f} ,ag}} + N_{{n_{f} ,bk}} .} \\ \end{array}$$

### Calculation of aggregation efficiency

Collisions are detected by monitoring potential overlaps between particles. Similar to three floc states, the formation of flocs of given ${n}_{f}$ can also be classified into three categories by comparing flocs at the current time ($t$) and the previous time ($t-\Delta t$): (i) Aggregation by smaller flocs, ${n}_{f}\left(t\right)>{n}_{f}\left(t-\Delta t\right)$; (ii) Breakage from larger flocs, ${n}_{f}\left(t\right)<{n}_{f}\left(t-\Delta t\right)$; (iii) Unchanged, ${n}_{f}\left(t\right)={n}_{f}\left(t-\Delta t\right)$. The number of flocs formed by aggregation is named ${N}_{{n}_{f},tot}$. Similarly, by further tracking those flocs formed by aggregation from the current time ($t$) to the next output time ($t+\Delta t$), the number of stable aggregated flocs (${N}_{{n}_{f},s}$), which retains all the primary particles in the same floc for another $\Delta t$, can be obtained. We use the term “aggregation efficiency”, defined as

17 $$\begin{array}{*{20}c} {E_{c} = N_{{n_{f} ,s}} /N_{{n_{f} ,tot}} ,} \\ \end{array}$$

which represents the probability of two or more flocs stay together after successive time steps. In other words, it indicates the stability of flocs formed by aggregation.

### Largest fragment ratio

Similar to the use of mass ratio after and before breakup to study the breakup mode^17^, we use the ratio between the number of primary particle of the largest fragment after breakage ${n}_{f,lg}$ to the parent floc ${n}_{f}$ as

18 $$\begin{array}{*{20}c} {LFR = n_{f,lg} /n_{f} .} \\ \end{array}$$

Erosion occurs when parent flocs only lose a few particles, resulting in $LFR\sim 1$. When splitting occurs, the daughter flocs have comparable sizes with $LFR\sim 0.5$ if only consider binary breakup.

### The population balance equation (PBE)

To model the aggregation-breakup system, the population balance equation is often implemented as^68^

19 $$\begin{aligned} & \frac{{\partial n\left( {\upsilon ,{\varvec{x}},t} \right)}}{\partial t} - \frac{{\partial n\left( {\upsilon ,{\varvec{x}},t} \right)\left[ {u_{i} \left( {{\varvec{x}},t} \right) - \delta_{i3} W_{s} } \right]}}{{\partial x_{i} }} - \frac{\partial }{{\partial x_{i} }}\left( {\kappa \frac{{\partial n\left( {\upsilon ,{\varvec{x}},t} \right)}}{{\partial x_{i} }}} \right) \\ & \quad = \begin{array}{*{20}c} {\frac{1}{2}\mathop \smallint \limits_{0}^{\upsilon } n\left( {\upsilon - \upsilon^{\prime},{\varvec{x}},t} \right) n\left( {\upsilon^{\prime} ,{\varvec{x}},t} \right) Q\left( {\upsilon - \upsilon^{\prime},\upsilon^{\prime}} \right) d\upsilon^{\prime} + \mathop \smallint \limits_{\upsilon}^{\infty } \beta \left( {\upsilon ,\upsilon^{\prime}} \right){\Gamma }\left( {\upsilon^{\prime}} \right)n\left( {\upsilon^{\prime},{\varvec{x}},t} \right) d\upsilon^{\prime}} \\ \end{array} \\ & \quad \quad - \mathop \smallint \limits_{0}^{\infty } n\left( {\upsilon ,{\varvec{x}},t} \right) n\left( {\upsilon^{\prime},{\varvec{x}},t} \right) Q\left( {\upsilon ,\upsilon^{\prime}} \right) d\upsilon^{\prime} - {\Gamma }\left( \upsilon \right)n\left( {\upsilon ,{\varvec{x}},t} \right), \\ \end{aligned}$$

where $n(\upsilon ,{\varvec{x}},t)$ is the number density of particle with volume $v$ at time $t$ and location ${\varvec{x}}$. ${W}_{s}$ is the settling velocity and ${u}_{i}$ is the fluid velocity component in the *i*-th direction. $\kappa$ is the sum of the molecular and turbulent diffusivity, $\delta$ is the Kronecker delta. $Q$ is the aggregation kernel typically consisting of the collision frequency and efficiency. $\Gamma$ is the breakup rate and $\beta$ is the fragmentation distribution function describing the created number of daughter flocs of volume $\upsilon^{\prime }$ after the breakage of a mother floc of volume $\upsilon$. The first two terms on the right-hand side (RHS) are the construction terms that generate flocs of size $\upsilon$: (i) Construction by aggregation of smaller flocs and (ii) Construction by breakup from larger flocs, respectively. The third and fourth terms on the RHS are the destruction terms that consume flocs of size $\upsilon$: (iii) Destruction by aggregation into larger flocs and (iv) Destruction by breakup into smaller flocs.

## Supplementary Information

Supplementary Information.

## Data Availability

All data generated or analyzed during this study are available upon request to the corresponding author.
