# Supplementary material for: Persistent reshaping of cohesive sediment towards stable flocs by turbulence
Source: Sci Rep. 2023 Jan 31;13:1760. doi: 10.1038/s41598-023-28960-y (PMC9889388; doi:10.1038/s41598-023-28960-y)
Supplement: Supplementary file 1 — Supplementary Information. [file 41598_2023_28960_MOESM1_ESM.docx]

SUPPLEMENTARY INFORMATION

# Supplementary Tables

| **Case** | $\boldsymbol{R}\boldsymbol{e}_{\boldsymbol{\lambda}}$ | $\boldsymbol{N}_{\boldsymbol{p}}$ | $\boldsymbol{\phi}$ | $\boldsymbol{D}_{\boldsymbol{p}}$ | $\boldsymbol{\eta}$ | $\boldsymbol{\lambda}$ | $\boldsymbol{G}$ | $\boldsymbol{\gamma}_{\boldsymbol{JKR}}$ | $\boldsymbol{\gamma}_{\boldsymbol{A}_{\boldsymbol{H}}}$ | $\boldsymbol{Co}$ |
| --- | --- | --- | --- | --- | --- | --- | --- | --- | --- | --- |
| S1 | 33 | 50,000 | $4.09\times{10}^{-4}$ | 0.02 | 0.053 | 0.63 | 1.60 | $2\times{10}^{-4}$ | $1\times{10}^{-3}$ | 0.54 |
| S2 |  |  |  |  |  |  |  | $4\times{10}^{-3}$ | $8\times{10}^{-3}$ | 5.39 |

**Supplementary Table S1 | Summary of normalized parameters of the direct numerical simulation of turbulence and the properties of primary particles.** $Re_{\lambda}$is the Taylor Reynolds number, $\eta$ is the Kolmogorov length scale and $\lambda$ is the Taylor microscale. G is the shear rate. $N_{p}$ is the total number of primary particles and $\phi$ is the corresponding solid volume fraction. All particles have identical diameter $D_{p}=0.02$. $\gamma_{JKR}$ is the particle surface energy density for the Johnson-Kendell-Robert (JKR) contact model, while $\gamma_{A_{H}}$is the particle surface energy density for the van der Waals force.

| **Category** | $\boldsymbol{L}_{\boldsymbol{a}}\boldsymbol{\&}\boldsymbol{L}_{\boldsymbol{b}}$ | $\boldsymbol{L}_{\boldsymbol{b}}\boldsymbol{\&}\boldsymbol{L}_{\boldsymbol{c}}$ |
| --- | --- | --- |
| Equant | $L_{b}$< $L_{a}$ < ${rL}_{b}$ | $L_{c}$ < $L_{b}$ < ${rL}_{c}$ |
| Prolate | $L_{a}$ > ${rL}_{b}$ | $L_{c}$ < $L_{b}$ < ${rL}_{c}$ |
| Oblate | $L_{b}$ < $L_{a}$ < ${rL}_{b}$ | $L_{b}$> ${rL}_{c}$ |
| Bladed | $L_{a}$ > ${rl}_{b}$ | $L_{b}$> ${rL}_{c}$ |

**Supplementary Table S2 | Zingg’s shape classification.** Particle shape can be described simply in terms of deviation from equancy in two respects, elongation (${L_{a}}/{L_{b}}$) and flatness (${L_{b}}/{L_{c}}$). $r$ is set to be 1.5 according to Zingg (1935) The triaxial size of the flocs satisfies $L_{c}<L_{b}<L_{a}$ (Methods). Flocs can be divided into four classes named equant, prolate, oblate and bladed with descending sphericity.
